# The Cause and Prevention of Death from Chloroform

**Published:** 1852-08

**Authors:** 


*The Cause and Prevention of Death from Chloroform.*—Dr. Snow said that when dogs, cats, or rabbits were made to breathe air containing from three to five per cent of vapor of chloroform till they died—a process which occupied generally from ten to fifteen minutes—the heart continued to act for a minute or so after the breathing had ceased, as he had ascertained by means of the stethescope, and then, in some instances, the animal gave a few gasping inspirations about the time when the heart was ceasing to act, which had the effect of restoring it to life. On the other hand, when such such animals were made to breathe air containing eight per cent or more of the vapor, death took place very suddenly, the respiration and the heart's action ceasing together. He had indeed performed three experiments in which the action of the heart stopped before the breathing. In experiments with sulphuric ether, the action of the heart always survived the respiration, as air containing fifty per cent of ether vapor was not more powerful than when it contained but five per cent of vapor of chleroform. Ether could, however, be made to act directly on the heart, by continuing to exhibit it by artificial respiration after the natural breathing ceased.

He believed that no accident had occurred from the continued exhibition of chloroform vapor, well diluted with air. In the fatal cases which had happened, death had taken place suddenly, by the way of syncope, showing that the heart had been paralysed by the action of vapor constituting not less than eight or ten per cent of the air inspired just before death. He enumerated all the deaths which he considered to have been caused by the administration of chloroform. They were eighteen in number. In sixteen of these, the agent was exhibited on a handkerchief, or towel, or piece of lint, and in the two cases in which some form of inhaler was employed, it was not used by a medical man. The subjects of these accidents had enjoyed a greater amount of general health than the average of those who had taken chloroform; none of them were children or old people, and the operations which were intended or had been commenced, were, with two or three exceptions, of a triflng nature. He considered the reason of this to be, that under such circumstances the same amount of care was not always employed as in more serious cases. There were two methods of ensuring the dilution of vapor of chloroform with atmospheric air, to such an extent that death could not occur without giving sufficient warning to allow of accidents being prevented by ordinary attention and skill.

The first and best of these methods was, to exhibit pure chloroform by means of a suitable inhaler; the other method was, to dilute the chloroform with rectified spirit of wine, before pouring it on a handkerchief or sponge. One part, by measure, of chloroform, to two of spirit, which constituted the strong chloric ether of Warren, of America, answered very well; but he (Dr. Snow) gave the preference to equal parts, by measure, of chloroform and spirit, which he was in the habit of applying by means of a sponge, in operation on the face, when he could not employ the inhaler. The best means to be employed in case of impending death from chloroform, was artificial respiration. He believed, from experiments he had performed on animals, that if it were instituted within half a minute of the apparent death of the patient, it would, in the greater number of cases, be attended with success. If this measure did not very quickly restore the patient, it would be advisable to open the external jugular vein, whilst still continuing the artificial breathing, in order to relieve the distension of the right cavities of the heart, which in these cases begins to take place as soon as its action ceases.

Dr. Crisp had found twenty cases of death from chloroform had been published. He had placed them in a tabular form, and enumerated them to the Society. His opinion respecting chloroform, deduced from those cases and his own observation was, that we could not employ it, even to healthy individuals, without some amount of danger. He differed from Dr. Snow in his opinion regarding the impossibility of robberies being effected by the agenby of chloroform, as it might easily be applied to half inebriated persons. An important question was, what are the ultimate effects of chloroform on those who take it in surgical operations? This, he contended, could only be answered by 15,000 or 20,000 cases.

Dr. Theophilus Thompson inquired the experience of Dr. Snow respecting the effects of chloroform on the system in certain cases in which the change effected was often durable or important; in fact, a train of symptoms indicating more or less congestion of the brain, and lasting for many days, or even longer.

Mr. Bullock said that the effects of chloroform were materially modified by its purity; much impure chloroform was manufactured. He had seen it administered to a patient, who was three hours under its influence, without any bad effects.

Mr. Richardson believed that cases of death from chloroform had occurred which had not been alluded to by Dr. Snow, and he mentioned one in particular which had taken place in Bruges. In deaths from chloroform in animals, he found the right side of the heart congested, and left auricle contracted; for thirty-five minutes after death in one case—the peristaltic action of the bowels also continued after death.

Dr. Camps considered that the influence of idiosyncracy should not be overlooked in estimating the effects of chloroform in particular instances. He believed that the first effects of chloroform were principally on the nervous system.

Mr. Barlow thought that the danger of administering chloroform by sprinkling it on a handkerchief, had been overrated. If there were not too much chloroform used, if the handkerchief were not placed too near, and there were not too many bystanders, and the patient were properly watched, he saw no reason to fear the administration of chloroform without an instrument. Tne proper note of the effects of chloroform during its administration was most essential. One person should always be watching for these bad effects. He had never seen a patient in danger, so long as the iris remained contracted; but if the iris dilated, the inspiration became difficult and the pulse flagged, the chloroform should be immediately discontinued. The deaths in some cases, he believed, had arisen from the desire, on the part of the operator, to administer the agent too rapidly. When death occurred, he believed it was from the heart becoming suddenly affected. The history of cases, however, in which the use of chloroform had been attended with a fatal result, had not, in some cases, been sufficiently stated for us to form a correct opinion upon them; often the previous condition of the patient was not mentioned. In some of the cases better recorded, a defective condition of the structure of the heart was present. What should we do in a case of poisoning by chloroform? We should employ all the means recommended by Dr. Snow, and more espicially resort to artificial respiration early.

Dr. Sibson said, that owing to the researches of Dr. Snow, we were now in a condition to determine the exact quantity of chloroform which was admitted into the system. He believed that in cases of death from chloroform, it was the heart that was at fault; the symptoms presenting themselves, such as sudden pallor, etc., showed this to be the case. He did not agree with Mr. Barlow, that it was necessary to stop the chloroform when the iris contracted, as in some cases, as of dislocation and hernia, it was necessary to carry the use of the agent beyond this point, which might be done with safety under proper precautions. With respect to the use of chloroform in neuralgia, it was not necessary to carry it to the extent of unconsciousness; anæsthesia was produced before this. With respect to the number of deaths which had occurred from the use of chloroform, he thought this was little, in comparison with the number of persons who had been relieved by its use.

Mr. C. Clark inquired the experience of members in respect to the employment of chloroform in midwifery.

Dr. Murphy advocated its use in midwifery in suitable cases, and with proper precautions. Deaths from chloroform were usually the result of carelessness in its administration—deaths resulting from the employment of too concentrated a dose, which acted suddenly upon the heart.

Dr. Chowne spoke at some length on the importance of the subject, and the circumspection which was necessary to be observed in the employment of chloroform. With respect to its use in midwifery, he thought many disasters had resulted from it, not only as referred to immediate, but to after consequences. Many cases had occurred which had not been published. He cautioned the members respecting the employment of chloroform, to which he was no enemy, but he was desirous to see its use accompanied with the greatest caution.

Dr. Snow said in reply, that the difference between the number of the deaths from chloroform in the list he had given, and in other lists, arose chiefly from the circumstance that he had excluded some deaths which had been attributed to this agent, but were due, in his opinion, to other causes. The case of the child mentioned by Dr. Crisp, for instance, occurred in Germany, during the excision of a very large nævus on the side of the face and neck, which surgeons, who had seen the case previously, were afraid to meddle with. The operation lasted eighteen minutes, only nine drops of chloroform were applied altogether, and none at all during the last eight minutes. Death was evidently due to syncope from the effects of the operation. As regarded the cases in which death happened during a second attempt to render the patient insensible by chloroform, they could not be attributed to the cumulative effects of the vapor which had been inhaled during the first process; for chloroform could not accumulate for more than twenty or thirty seconds; after this time it began to be exhaled again. These cases clearly illustrated the uncertainty and irregularity of the means which had been employed in administering the chloroform, and showed that the accidents were not due to any peculiar susceptibility to its effects on the part of the patient, who could not have two different idiosyncrasies nearly at the same time; first, a want af susceptibility, and a few minutes or half an hour afterwards, a greater susceptibility than usual.

In reply to Dr. Theophilus Thompson, Dr. Snow stated, that he had not met with any unpleasant sequalæ which he considered to be the effects of chloroform, except sickness, which had in a few cases been troublesome for two or three days, and hysteria, the latter of which might certainly occur from an operation without chloroform. If depression existed from the long-continued administration of chloroform, it should be removed by warmth and cordials.—*London Lancet.*

